# Response of *Daphnia*'*s* Antioxidant System to Spatial Heterogeneity in Cyanobacteria Concentrations in a Lowland Reservoir

**DOI:** 10.1371/journal.pone.0112597

**Published:** 2014-11-07

**Authors:** Adrianna Wojtal-Frankiewicz, Joanna Bernasińska, Piotr Frankiewicz, Krzysztof Gwoździński, Tomasz Jurczak

**Affiliations:** 1 Department of Applied Ecology, University of Lodz, Lodz, Poland; 2 Department of Molecular Biophysics, University of Lodz, Lodz, Poland; CEA-Saclay, France

## Abstract

Many species and clones of *Daphnia* inhabit ecosystems with permanent algal blooms, and they can develop tolerance to cyanobacterial toxins. In the current study, we examined the spatial differences in the response of *Daphnia longispina* to the toxic *Microcystis aeruginosa* in a lowland eutrophic dam reservoir between June (before blooms) and September (during blooms). The reservoir showed a distinct spatial pattern in cyanobacteria abundance resulting from the wind direction: the station closest to the dam was characterised by persistently high *Microcystis* biomass, whereas the upstream stations had a significantly lower biomass of *Microcystis*. Microcystin concentrations were closely correlated with the cyanobacteria abundance (r = 0.93). The density of daphniids did not differ among the stations. The main objective of this study was to investigate how the distribution of toxic *Microcystis* blooms affects the antioxidant system of *Daphnia*. We examined catalase (CAT) activity, the level of the low molecular weight antioxidant glutathione (GSH), glutathione S-transferase (GST) activity and oxidative stress parameters, such as lipid peroxidation (LPO). We found that the higher the abundance (and toxicity) of the cyanobacteria, the lower the values of the antioxidant parameters. The CAT activity and LPO level were always significantly lower at the station with the highest *M. aeruginosa* biomass, which indicated the low oxidative stress of *D. longispina* at the site with the potentially high toxic thread. However, the low concentration of GSH and the highest activity of GST indicated the occurrence of detoxification processes at this site. These results demonstrate that daphniids that have coexisted with a high biomass of toxic cyanobacteria have effective mechanisms that protect them against the toxic effects of microcystins. We also conclude that *Daphnia*'s resistance capacity to *Microcystis* toxins may differ within an ecosystem, depending on the bloom's spatial distribution.

## Introduction

Planktivorous zooplankton are one of the groups most affected by the mass development of toxic cyanobacteria in inland waters [1]. Specifically, the large-bodied, efficient grazer *Daphnia* usually exhibits slower growth rates and decreased survival and reproduction in the presence of cyanobacteria [2]–[4]. However, in recent years, it has been observed that the sensitivity of *Daphnia* to cyanobacteria depends on the species and even varies among clones [5]–[7]. An increasing number of publications have shown that *Daphnia* populations can evolve mechanisms that allow them to coexist with toxic cyanobacteria [8]–[10], [6]. Such resistance results from genetic changes that result in the local co-adaptation of *Daphnia* to cyanobacterial toxins [11], [12]. The sensitivity of daphniids to cyanotoxins is most striking in species or clones that are isolated from distinctive habitat types, ecosystems with different trophy and abundances of toxic strains of cyanobacteria [13], [5], [14]. Little is known about how *Daphnia* sp. respond to spatial differences in cyanobacteria abundance within an ecosystem. Instead, previous research has focused on asynchrony in the zooplankton – the spatial distribution of cyanobacteria and the formation of the “refuge sites” that allow large grazers to persist during blooms [15], [16].

The main objective of this study was to investigate how the antioxidant system in *Daphnia longispina* (O. F. Müller) responds to the spatial distribution of toxic *Microcystis aeruginosa* (Kutzing) blooms within a lowland reservoir. Our previous research indicated that daphniids that had coexisted with high concentrations of microcystins in the environment had effective mechanisms to protect them against the accumulation and toxic effect of these metabolites [17]. On the basis of those results, we hypothesise that the oxidative stress of *D. longispina* in the sites with high toxic cyanobacteria abundance will be relatively low compared to sites with less biomass of *Microcystis*.

## Materials and Methods

### Ethics statement

No specific permits were required for the field studies described herein. There was no activity involving endangered or protected species in this study.

### Study Site

**Figure 1:**
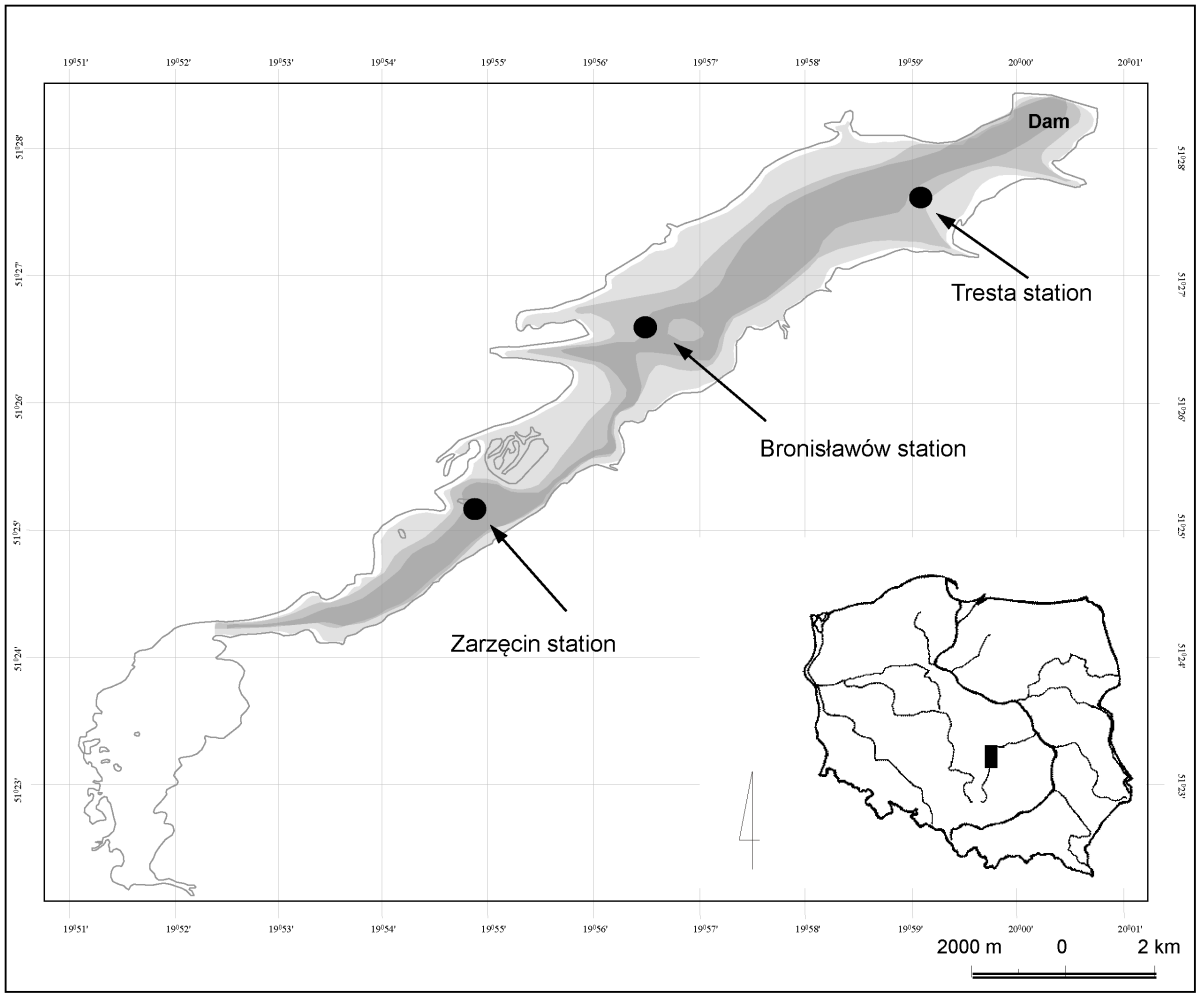
Study site. Map of the Sulejow Reservoir with sampling sites.

Sulejow Reservoir is a 39-year-old lowland dam reservoir situated on km 138.9 of the Pilica River (the Vistula River catchment) in central Poland (Fig. 1). Its maximum length is 15.5 km, and its maximum width is 2.1 km. At its maximum capacity (75×10^6^ m^3^), the reservoir covers 1980 ha, with a mean depth of 3.3 m and a maximum depth of 11 m. The mean water retention time in the reservoir is 30 days [18]. The reservoir subcatchment is mainly covered by agricultural land (50% arable land, 13% meadows and pastures, 1% orchards) and forests (31%). The length of the shoreline is approximately 54 km. The Sulejow Reservoir is a eutrophic ecosystem with annual cyanobacterial blooms [17], [18], [19]. The dominant species of bloom-forming cyanobacteria is *Microcystis aeruginosa*, which produces microcystin-LR, -YR and RR [20], [21]. The genus was determined by verifying the accuracy of the amplification products obtained for the 16S rRNA gene and mcyA gene, which are specific to the *Microcystis* genera. In all the analysed samples, we found homology (99–100%) for *M. aeruginosa* NIES-843 [22].

Studies were conducted at three sampling stations in the Sulejow Reservoir in 2012: Tresta (TR), Bronisławów (BR) and Zarzęcin (ZA). The TR station is located in the lower section near the dam. The BR is located in the middle section of the reservoir in front of the former water pump station, and the ZA station is located in the upper part of the reservoir near the backwater (Fig. 1). The fieldwork was conducted in four periods: before cyanobacterial blooms (beginning of June) and during blooms (July–September). The sampling dates were established according to the results of monitoring of the Sulejow Reservoir, which has been performed weekly for eighteen years from April to October by the Department of Applied Ecology in the University of Lodz. Thus, the sampling was conducted on June 4^th^, July 2^nd^, August 21^st^ and September 26^th^. However, in June we performed the fieldwork only on two sites at opposite ends of the reservoir, Tresta and Zarzęcin, due to the clear water phase in the reservoir and homogenous physical, chemical and biological conditions at BR and ZA (data not shown).

To confirm the pattern observed in 2012, additional samples were collected at TR, BR and ZA on 11^th^ September 2014.

### Plankton collection, preparation and identification

Zooplankton samples were collected from a 0.5-m depth using a 64-µm mesh. A net with a diameter of 0.25 m was dragged by a boat for 10 min with a speed of 0.5 m s^−1^, which resulted in sieving approximately 15,000 L of water. During the most intensive cyanobacterial blooms (in August and September), zooplankton were collected using a 5-L sampler (with many repetitions) at a 4-m depth due to the high concentration of *Microcystis aeruginosa* in the surface water layer. This procedure was feasible because regular mixing of the reservoir's water caused unification of the physical and chemical conditions in the water column, which was controlled by the YSI Professional Plus multisensors during sampling (data not shown).

In the laboratory, the samples were placed into a 2-L glass separator and repeatedly washed by water to separate the zooplankton from the phytoplankton. Next, individuals of *Daphnia* sp. were selected under a Nikon 102 microscope (magnification of ×40–60) with a pipette and placed into a vessel with distilled water. Then, *Daphnia* sp. individuals were gravitationally filtrated (without using a pump) on Whatman GF/C filter paper. The animals were gently removed from the filters with a needle and separated into nine Eppendorf test tubes (approximately 500–600 daphniids per each tube). The test tubes were frozen at −70°C before the chemical analyses.

To identify the zooplankton species and determine their density, 35 L of water was filtered using a 64 µm mesh net, and the samples were concentrated to 10 ml and preserved in a 4% Lugol's solution. Zooplankton taxa were distinguished under a Nikon 115 microscope (magnification of ×100–200). The *Daphnia* species collected were morphologically identified using Benzie [23].

Cyanobacteria species composition (qualitative analyses) was examined in 10 L^−1^ water samples taken on each sampling occasion. The water samples for phytoplankton estimation were preserved in a Lugol's solution and sedimented in the laboratory. Algae were counted using a Fusch-Rosenthal counting cell. At least 400 cells or colonies were counted to reduce the error to less than 10% (P = 0.05). The collected material was morphologically analysed according to Starmach [24], Komarek [25], and Komarek and Anagnostidis [26].

### Estimation of cyanobacteria abundance by chlorophyll *a* concentration

The concentration of chlorophyll *a* (µg L^−1^) was measured immediately after sampling in a 1-L integrated water sample using a bbe Algae Online Analyser (AOA, Version 1.5 E1, bbe-Moldaenke company Kiel, Germany). The measurement principle of the bbe AOA is based on the determination of the fluorescence spectrum and the fluorescence kinetics of the algae. By analysing the interaction of chlorophyll *a* with other pigments, AOA discriminates four main groups of algae (green algae, cyanobacteria, diatoms and cryptophytes). This method is recognised as a reliable on-line analysis for chlorophyll *a* measurements [27] and as a useful tool for monitoring the phytoplankton community composition, particularly as an early warning system for the detection of harmful algal blooms [28]. The AOA fluorometer has been previously tested as an early warning method for cyanobacterial blooms in the Sulejow Reservoir. The results of these studies demonstrated a significant positive correlation (r = 0.68, n = 46, *P*<0.05) between cyanobacterial biovolume, as determined by cell counts, and cyanobacterial chlorophyll *a*, as measured by the AOA [29].

### Microcystin concentrations – sample preparation and analyses

Samples of water containing cyanobacteria were stored in dark glass bottles of 1-L volume and transported to the laboratory for analyses immediately after sampling.

The microcystins (MCs) were analysed in two fractions: the first was dissolved in water (extracellular) and the second in cell-bound form in suspended matter (intracellular). For the analysis of microcystins in suspended matter, the water samples were filtered on Whatman GF/C filter paper and dried to estimate the dry biomass of cyanobacteria. The filters were then stored at −20°C until future use. Microcystins in the suspended material were extracted in 75% aqueous methanol. The samples were sonicated for 30 s in a Misonix ultrasonicator (Farmingdale, NY, USA) equipped with an ultrasonic probe (100 W, 19 mm diameter with “spike”) and a liquid processor XL. The extracts were then centrifuged twice at 11000×*g* for 10 min at 4°C in an Eppendorf 5804 centrifuge (Hamburg, Germany). The supernatants were collected and evaporated in a SC110A Speedvac Plus (ThermoSavant, Holbrook, NY, USA). Before the High Performance Liquid Chromatography (HPLC) analysis, the samples were redissolved in 1 ml of 75% aqueous methanol and filtered through a Gelman GHP Acrodisc 13-mm syringe filter with a 0.45-µm GHP membrane and a minispike outlet (East Hills, NY, USA).

For dissolved microcystins, 1-L samples of filtered water were concentrated in Baker C_18_ solid phase extraction (SPE) cartridges (Deventer, Netherlands; sorbent mass: 500 mg). The microcystins were eluted from the C_18_ cartridges with 3 ml of 90% aqueous methanol containing 0.1% trifluoroacetic acid (TFA). The eluates were then evaporated to dryness, and the samples were redissolved in 1 ml of 75% aqueous methanol before HPLC analysis.

The samples were analysed using an Agilent 1100 series (Waldbronn, Germany) HPLC comprising a quaternary pump, an autosampler, a thermostated column compartment and a diode-array detector. Chromatographic separation was achieved on a Merck Purospher Star RP-18e column (55×4 mm; 3 µm) with a C_18_ guard column (4×4 mm). A determination of microcystins by HPLC-DAD was performed using a gradient mobile phase of H_2_O +0.05% TFA (eluent A) and acetonitrile (ACN) +0.05% TFA (eluent B) and diode-array detection at 200–300 nm. The linear gradient conditions were as follows: 25% B at 0.0 min, 70% B at 5.0 min, 70% B at 6.0 min and 25% B at 6.1 min. The sample volume was 20 µl, the flow rate was 1 ml min^−1^ and the column temperature 40°C. The microcystins in the cyanobacterial extracts were identified using the microcystin standards MC-LR, MC-RR and MC-YR, with their characteristic absorption spectra and retention times. Microcystins MC-LR, MC-RR and MC-YR are the main microcystins detected in Polish freshwaters [30]. Three microcystin standards from seven commercially available products from Calbiochem (La Jolla, CA, USA) were used.

### Determination of thiobarbituric reactive substances

*Daphnia* sp. individuals (wet mass 0.06–0.16 g) were placed at a 10% w/v ratio into a 100-mM sodium phosphate buffer with a pH 7.4 with 100 mM KCl and 1 mM EDTA on ice.

Homogenisation using a CAT X-120 knife homogeniser was performed on ice at 2000 rpm for 2 min, and the homogenates were then centrifuged at 10,000×*g* for 10 min (4°C). The supernatants were immediately used for a cellular lipid peroxidation (LPO) estimation. Lipid peroxidation was measured using a thiobarbituric acid reactive substances (TBARS) assay [31] with modifications [32]. The concentration of the thiobarbituric acid reactive substances is an index of lipid peroxidation and oxidative stress. The assay was monitored for the appearance of conjugated complexes of thiobarbituric acid, mainly malondialdehyde (MDA), which is the end product of LP at 532 nm. The MDA levels were calculated using the MDA extinction coefficient, 156 L mmol^−1^ cm^−1^, and expressed as nanomoles per milligram of protein (nmol/mg protein).

### Determination of glutathione content

*Daphnia* sp. individuals (wet mass 0.06–0.16 g) were placed in a 10% w/v ratio in homogenisation buffer (154 mM KCl, 5 mM diethylenetriaminepentaacetic acid (DTPA) and 0.1 M potassium phosphate buffer, pH 6.8) and homogenised on ice at 2000 rpm for 2 min. An aliquot was removed for protein determination using the Lowry method [33]. The remainder of the homogenate was mixed with a cold acid buffer containing 40 mM HCl, 10 mM DTPA, 20 mM ascorbic acid and 10% trichloroacetic acid (TCA) at a ratio of 1∶1. The suspension was centrifuged at 13,000×*g* for 10 min (4°C). The supernatants were immediately used for further determination of the glutathione (GSH) content.

The GSH was estimated according to the fluorescence assay of Senft et al. [34] using o-phthalaldehyde (OPA). In this method, OPA, which has a low fluorescence background, reacts only with GSH to generate a strong fluorescence so that GSH can be specifically quantified. The OPA-derived fluorescence was measured at 365 nm excitation and 430 nm emission. The glutathione concentration was calculated using the calibration curve for different concentrations of reduced glutathione, as a standard, and expressed as nanomoles per milligram protein (nmol/mg protein).

### Determination of glutathione S-transferase activity

Glutathione S-transferase (GST) activity was determined in September 2014 using the method of Habig et al. [35] by evaluating the increase in absorbance at 340 nm due to the formation of the conjugate of the 1-chloro-2,4-dinitrobenzene (CDNB) substrate in the presence of reduced glutathione (GSH). The reaction mixture was prepared by mixing 1.5 ml sodium phosphate buffer 0.1 M pH 6.5, 0.2 ml GSH 9.2 mM, 0.02 ml CDNB 0.1 M and 0.1 ml of the sample. The absorbance was measured spectrophotometrically at 340 nm and +25°C.

### Determination of catalase activity

*Daphnia* sp. individuals (wet mass 0.06–0.16 g) were placed in a 10% w/v ratio in 100 mM sodium phosphate buffer, pH 7.4, with 100 mM KCl and 1 mM EDTA on ice. Homogenisation was performed on ice at 2000 rpm for 2 min, and the homogenates were then centrifuged at 10,000×*g* for 10 min (4°C). The supernatants were immediately used for the determination of catalase activity. The activity of catalase (CAT) was measured spectrophotometrically according to Aebi [36]. The method is based on the decomposition of hydrogen peroxide as indicated by decreased absorbance at 240 nm. The assay mixture consisted of homogenate, 50 mM potassium phosphate buffer pH 7.0 and 0.1% hydrogen peroxide in the final volume of 3 ml. The results of this enzymatic assay were reported in units of CAT activity per milligram of protein (U/mg protein), where 1 U of CAT is defined as the amount of enzyme decomposing 1 µmol of H_2_O_2_ per minute.

### Determination of the protein concentration

The protein concentration was evaluated using the spectrophotometric method [33] with Folin's reagent. The amount of protein in each sample was estimated using the calibration curve for different concentrations of bovine albumin as a standard.

### Statistical methods

All the statistical analyses were conducted in Statistica 9.0 (StatSoft, Inc). To test for the effects of the season and sites on the analysed parameters, we used a two-way ANOVA with the months and sites as categorical factors and the *Microcystis* biomass and oxidative stress parameters in *Daphnia* sp. tissues as the dependent factors.

To test for the effect of sites on the analysed parameters within a given month, we applied a one-way ANOVA with the sites as categorical factors and the *Microcystis* biomass and the oxidative stress parameters in *Daphnia* sp. tissues as the dependent factors.

To determine the relationship between *Microcystis* biomass and microcystin concentration, a Pearson correlation coefficient *r* was calculated.

## Results

### Identification of *Daphnia* species and determination of their densities

Two species of *Daphnia* were identified in the samples from the Sulejow Reservoir: *D. longispina* (O. F. Müller) and *D. cucullata* (Sars). Throughout the sampling season, the numerical share of *D. longispina* in the samples was on average 90%±7.12%, whereas the numerical share of *D. cucullata* was on average only 10%±7.12%. Thus, we isolated the species from the samples and analysed the dominant species. The densities of *D. longispina* did not differ between the stations. However, the densities did vary by season, which resulted from typical annual dynamics of *Daphnia* populations. The details are presented in Table 1.

**Table 1:** Density of *Daphnia longispina* in the sites of the Sulejow Reservoir.

Date	TR	BR	ZA
04/06/2012	32	36	31
02/07/2012	85	73	76
21/08/2012	42	37	41
26/09/2012	25	31	29
11/09/2014	53	48	59

Density of *Daphnia longispina* [ind dm^−3^] in the three studied sites: Tresta (TR), Bronisławów (BR) and Zarzęcin (ZA). The sampling was conducted monthly between June and September of 2012, and on September of 2014.

### Assessment of Cyanobacteria abundance by measurement of chlorophyll *a*

**Figure 2:**
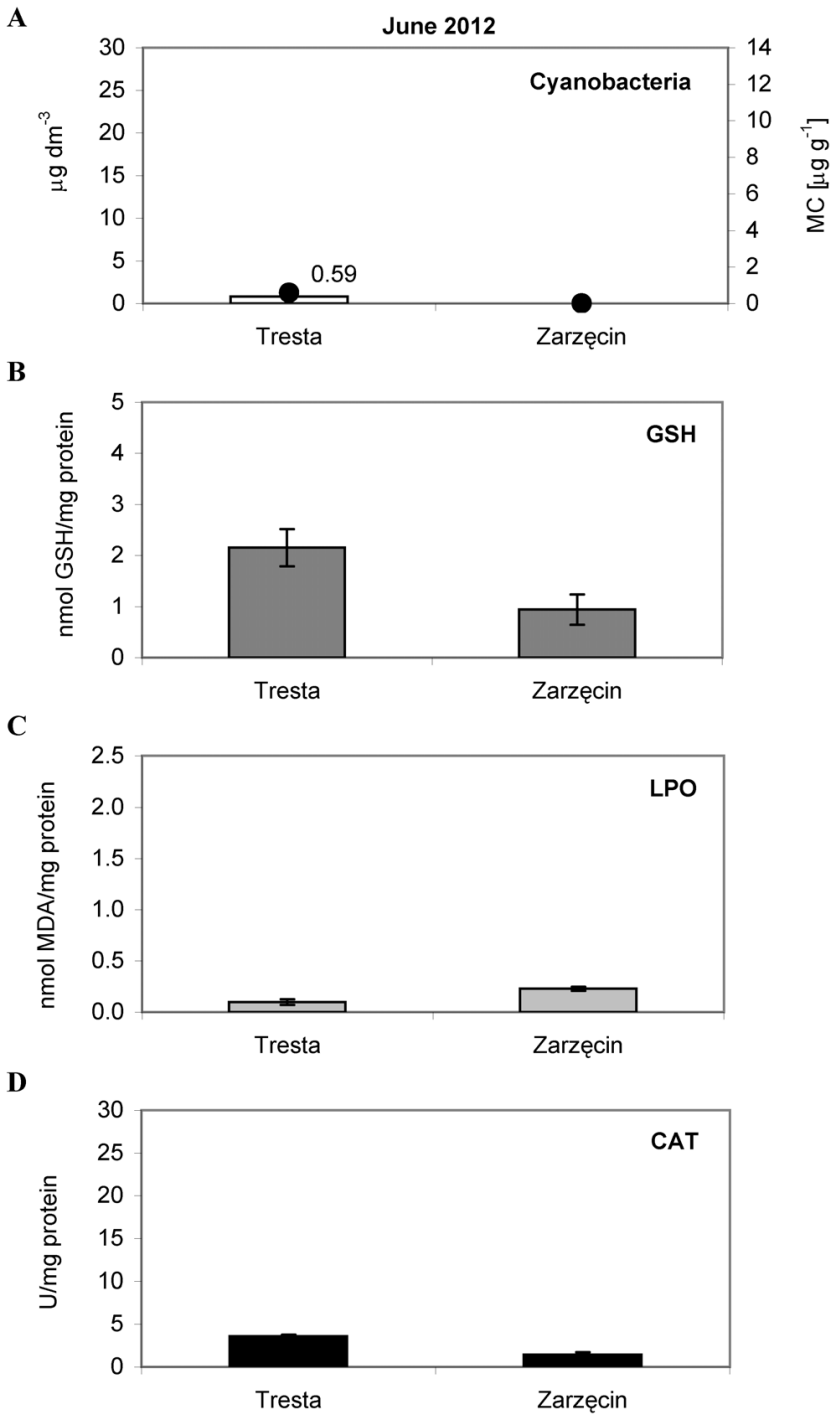
The cyanobacteria abundance and activity of the antioxidant parameters in *Daphnia* tissues, measured in June 2012. (A) The bars represent an average of three replicates (±S.D.) of cyanobacteria abundance (µg dm^−3^); the dark dots indicate the total concentration of microcystins LR and RR (µg g^−1^), (B) The bars represent an average of seven replicates (±S.D.) of glutathione (GSH) concentrations (nmol/mg protein), (C) The bars represent an average of seven replicates (±S.D.) of lipid peroxidation (LPO) (nmol/mg protein) and (D) The bars represent an average of six replicates (±S.D.) of catalase (CAT) activity (U/mg protein) in *Daphnia* tissues from the Sulejow Reservoir in 2012. The same letters above the bars indicate that the values did not differ significantly. Each panel of the figure includes the one-way ANOVA test results. Details concerning GSH, LPO and CAT data are presented in Tables S1, S2 and S3.

A qualitative analysis confirmed that the species of cyanobacteria occurring in all samples was *Microcystis aeruginosa*. In June, the chlorophyll *a* concentration for cyanobacteria in the Sulejow Reservoir was detected only at the TR station (Fig. 2A). The value was very low and averaged 0.80 µg L^−1^. During the summer, the chlorophyll *a* concentration increased, consistently reaching its highest values at the TR station (Table 2). The differences between months were also significant, and the test showed the following relationship between values of *Microcystin* abundance: July < August < September (see Table 2 for details of two-way ANOVA results).

**Table 2:** The two-way ANOVA test results.

Overall effect	df	F-ratio	P value	Tukey, posthoc
**CYANOBACTERIA**				
Months	2,18	73.31	<0.001	Jul <Aug <Sep
Sites	2,18	530.42	<0.001	TR> BR> ZA
Months x sites	4,18	90.93	<0.001	
**GSH**				
Months	2,54	75.39	<0.001	Aug <Sep <Jul
Sites	2,54	49.14	<0.001	TR <ZA <BR
Months x sites	4,54	24.01	<0.001	
**LPO**				
Months	2,54	67.53	<0.001	Aug <Sep <Jul
Sites	2,54	75.68	<0.001	TR <BR <ZA
Months x sites	4,54	32.30	<0.001	
**CAT**				
Months	2,45	828.23	<0.001	Aug <Sep <Jul
Sites	2,45	349.63	<0.001	TR <ZA <BR
Months x sites	4,45	180.54	<0.001	

Results of the two-way ANOVA test for the effects of month and site on the cyanobacteria abundance and antioxidant parameters measured in *Daphnia* tissues in 2012. GSH – glutathione, LPO – lipid peroxidation, CAT – catalase. Analysis do not include data from June.

**Figure 3:**
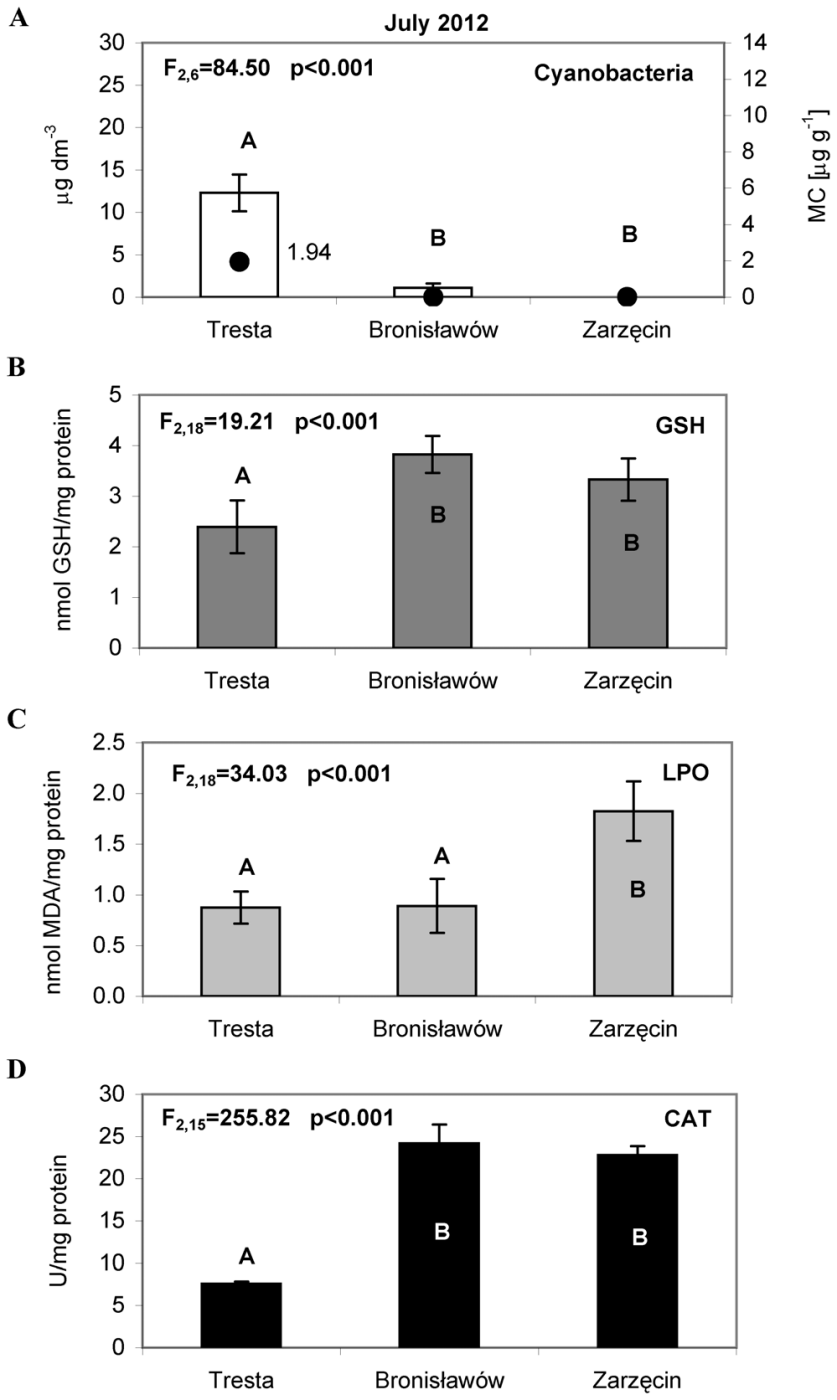
The cyanobacteria abundance and activity of the antioxidant parameters in *Daphnia* tissues, measured in July 2012. (A) The bars represent an average of three replicates (±S.D.) of cyanobacteria abundance (µg dm^−3^); the dark dots indicate the total concentration of microcystins LR and RR (µg g^−1^), (B) The bars represent an average of seven replicates (±S.D.) of glutathione (GSH) concentrations (nmol/mg protein), (C) The bars represent an average of seven replicates (±S.D.) of lipid peroxidation (LPO) (nmol/mg protein) and (D) The bars represent an average of six replicates (±S.D.) of catalase (CAT) activity (U/mg protein) in *Daphnia* tissues from the Sulejow Reservoir in 2012. The same letters above the bars indicate that the values did not differ significantly. Each panel of the figure includes the one-way ANOVA test results. Details concerning GSH, LPO and CAT data are presented in Tables S1, S2 and S3.

**Figure 4:**
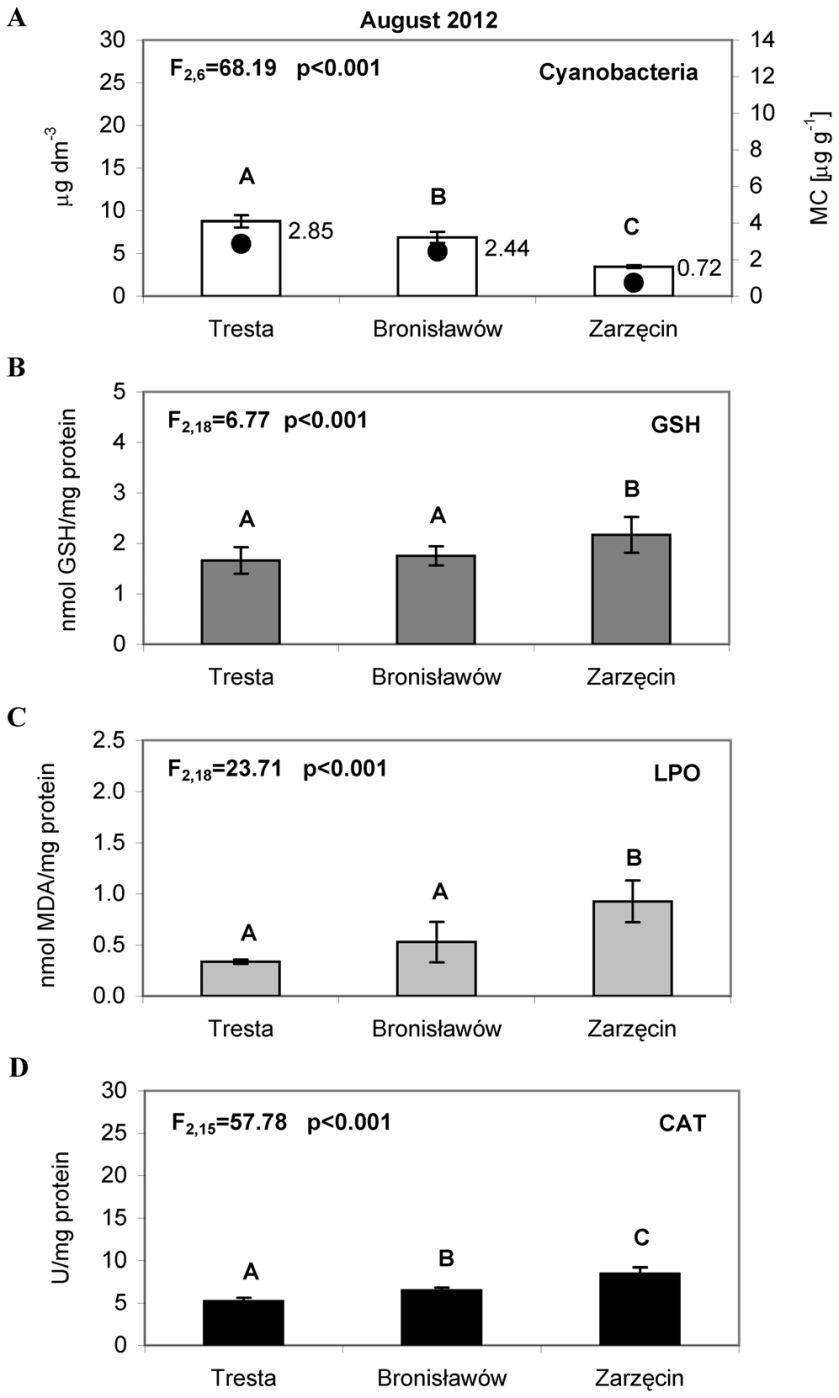
The cyanobacteria abundance and activity of the antioxidant parameters in *Daphnia* tissues, measured in August 2012. (A) The bars represent an average of three replicates (±S.D.) of cyanobacteria abundance (µg dm^−3^); the dark dots indicate the total concentration of microcystins LR and RR (µg g^−1^), (B) The bars represent an average of seven replicates (±S.D.) of glutathione (GSH) concentrations (nmol/mg protein), (C) The bars represent an average of seven replicates (±S.D.) of lipid peroxidation (LPO) (nmol/mg protein) and (D) The bars represent an average of six replicates (±S.D.) of catalase (CAT) activity (U/mg protein) in *Daphnia* tissues from the Sulejow Reservoir in 2012. The same letters above the bars indicate that the values did not differ significantly. Each panel of the figure includes the one-way ANOVA test results. Details concerning GSH, LPO and CAT data are presented in Tables S1, S2 and S3.

**Figure 5:**
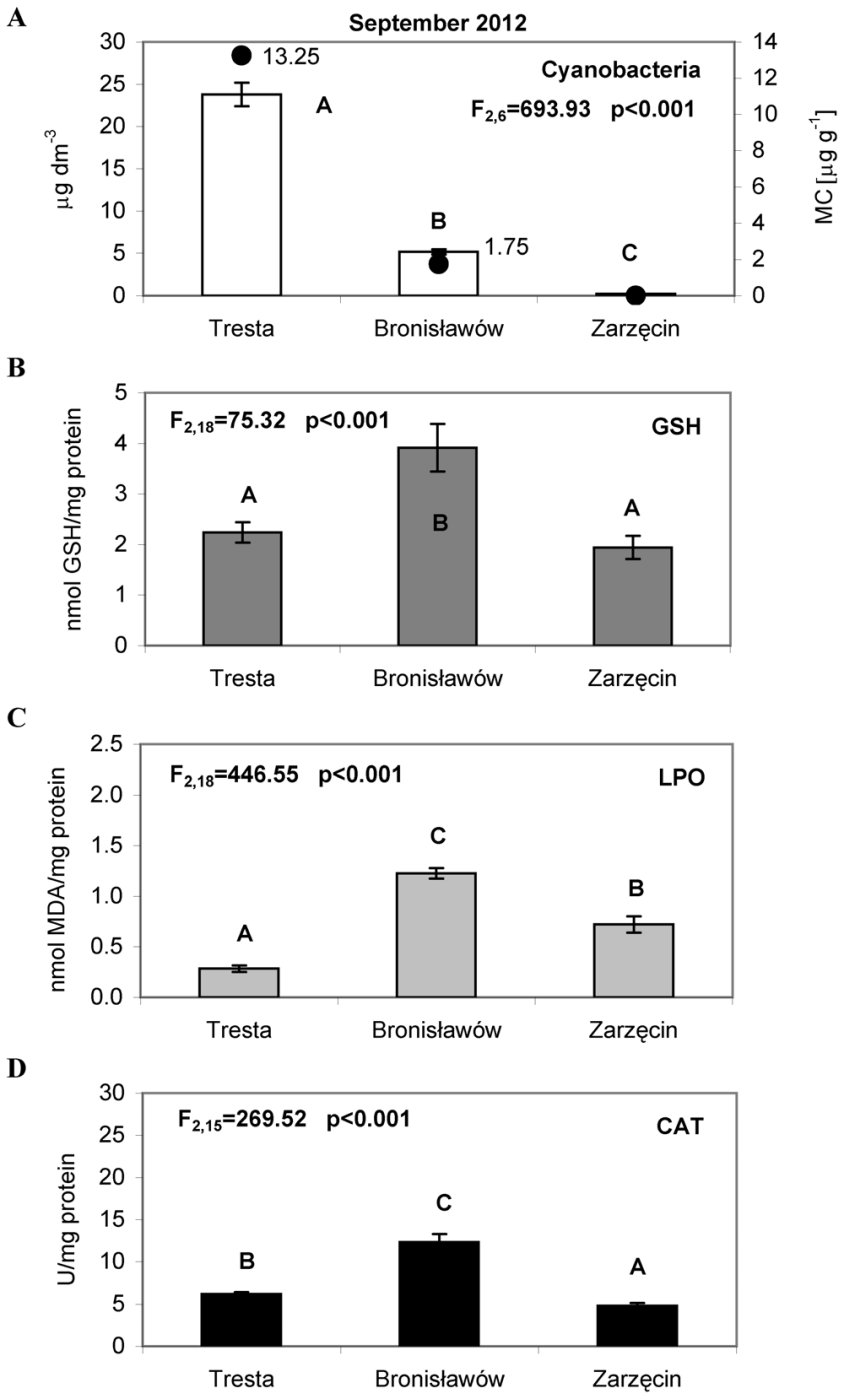
The cyanobacteria abundance and activity of the antioxidant parameters in *Daphnia* tissues, measured in September 2012. (A) The bars represent an average of three replicates (±S.D.) of cyanobacteria abundance (µg dm^−3^); the dark dots indicate the total concentration of microcystins LR and RR (µg g^−1^), (B) The bars represent an average of seven replicates (±S.D.) of glutathione (GSH) concentrations (nmol/mg protein), (C) The bars represent an average of seven replicates (±S.D.) of lipid peroxidation (LPO) (nmol/mg protein) and (D) The bars represent an average of six replicates (±S.D.) of catalase (CAT) activity (U/mg protein) in *Daphnia* tissues from the Sulejow Reservoir in 2012. The same letters above the bars indicate that the values did not differ significantly. Each panel of the figure includes the one-way ANOVA test results. Details concerning GSH, LPO and CAT data are presented in Tables S1, S2 and S3.

In July, the chlorophyll *a* concentrations for cyanobacteria were 12.27 µg L^−1^ at the TR station and 1.08 µg L^−1^ at the BR station (Fig. 3A). There were no cyanobacteria present at the ZA station. In August, the concentration of chlorophyll *a* for cyanobacteria was 8.75 µg L^−1^ at TR, 6.89 µg L^−1^ at BR, and 3.46 µg L^−1^ at ZA (Fig. 4A). During September, a substantial bloom of *M. aeruginosa* was observed at the TR station (23.77 µg L^−1^). At the other sites, the concentration of chlorophyll *a* for cyanobacteria was significantly lower, only 5.16 µg L^−1^ at BR and 0.23 µg L^−1^ at ZA (Fig. 5A). The analysis of the interaction plot indicated that there was a significant effect of the months × sites interaction, resulting mostly from high *Microcystis* abundance at the Tresta station in July and September.

**Figure 6:**
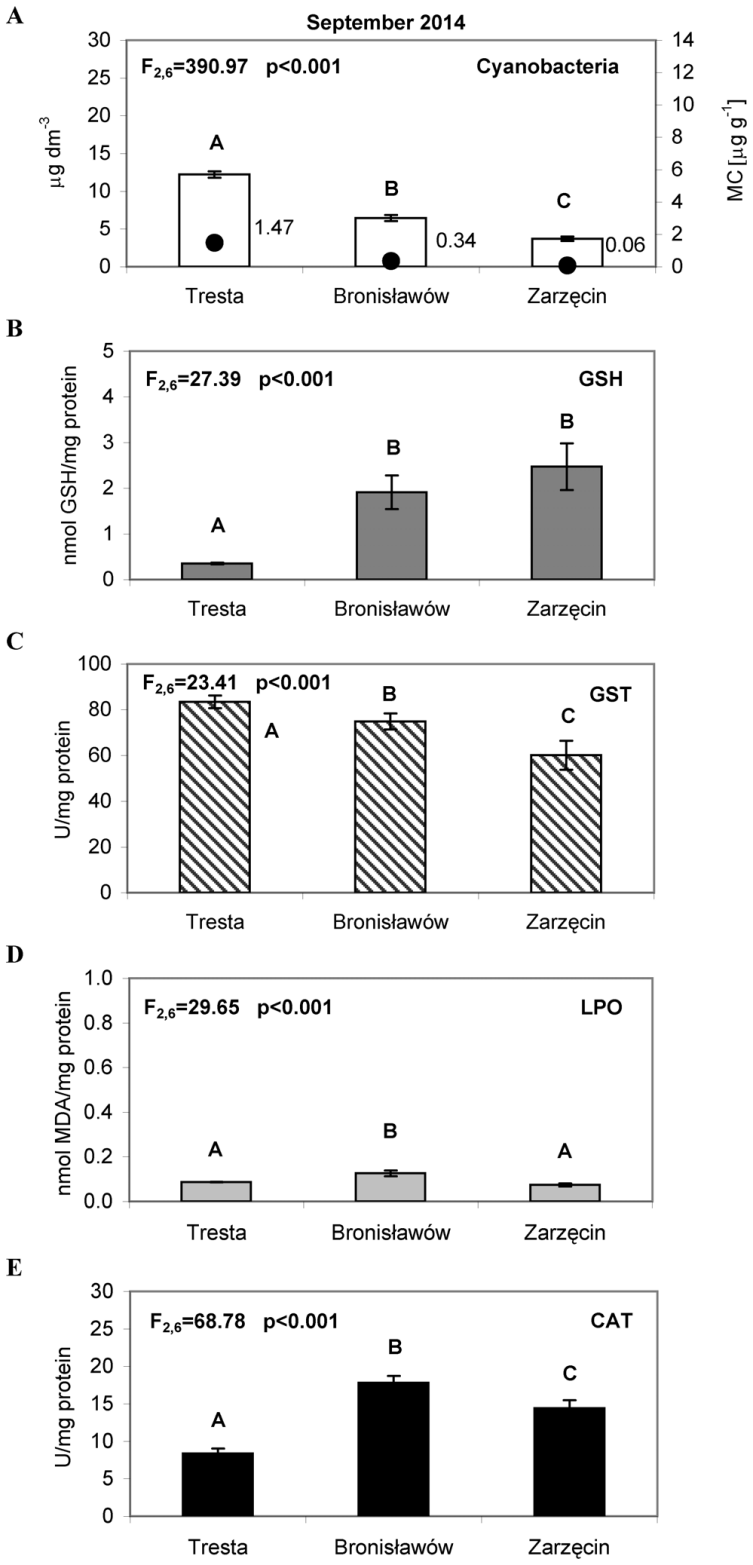
The cyanobacteria abundance and activity of the antioxidant parameters in *Daphnia* tissues, measured in September 2014. (A) The bars represent an average of three replicates (±S.D.) of cyanobacteria abundance (µg dm^−3^); the dark dots indicate the total concentration of microcystins LR, YR and RR (µg g^−1^), (B) The bars represent an average of three replicates (±S.D.) of glutathione (GSH) concentrations (nmol/mg protein), (C) The bars represent an average of three replicates (±S.D.) of glutathione S-transferase (GST) activity (U/mg protein) (D) The bars represent an average of three replicates (±S.D.) of lipid peroxidation (LPO) (nmol/mg protein) and (D) The bars represent an average of three replicates (±S.D.) of catalase (CAT) activity (U/mg protein) in *Daphnia* tissues from the Sulejow Reservoir in 2014. The same letters above the bars indicate that the values did not differ significantly. Each panel of the figure includes the one-way ANOVA test results. Details concerning GSH, GST, LPO and CAT data are presented in Tables S1, S2, S3 and S4.

In September 2014, the concentration of chlorophyll *a* for cyanobacteria was 12.21 µg L^−1^ at TR, 6.43 µg L^−1^ at BR, and 3.70 µg L^−1^ at ZA (Fig. 6A).

A one-way ANOVA indicated that the average values of *M. aeruginosa* abundance differed significantly between the sites during all the sampling periods (details on Figs. 2A–6A).

### Microcystin concentrations

In 2012, the microcystins LR and RR in samples from the Sulejow Reservoir were identified. An HPLC analysis detected the presence of microcystins in the cells (Fig. 2A–5A) but not in the water of the studied ecosystem. In June, the concentrations of MC-RR in the cells were 0.35 µg g^−1^, and the concentrations of MC-LR were 0.24 µg g^−1^ at the TR station. In July, the microcystins were still detected only at the TR station, and their concentration increased to 1.06 µg g^−1^ (MC-RR) and 0.88 µg g^−1^ (MC-LR). In August, both MC-RR and MC-LR were found at all three stations: MC-RR  = 1.67 µg g^−1^ and MC-LR  = 1.18 µg g^−1^ at TR; MC-RR  = 1.53 µg g^−1^ and MC-LR  = 0.91 µg g^−1^ at BR, and MC-RR  = 0.48 µg g^−1^ and MC-LR  = 0.24 µg g^−1^ at ZA. The highest values of microcystins at the TR station were detected in September (MC-RR  = 10.66 µg g^−1^ and MC-LR  = 2.58 µg g^−1^). At the BR station, MC-RR reached 1.02 µg g^−1^ and MC-LR reached 0.73 µg g^−1^. We did not find microcystins at ZA (Fig. 5A).

In September 2014, we identified the microcystins LR, YR and RR in the samples. The highest values of microcystins at the TR station were MC-RR  = 0.86 µg g^−1^, MC-YR  = 0.20 µg g^−1^ and MC-LR  = 0.41 µg g^−1^. At the BR station, MC-RR reached 0.19 µg g^−1^, MC-YR reached 0.05 µg g^−1^ and MC-LR reached 0.10 µg g^−1^. At the ZA station, only MC-RR was identified in an amount of 0.06 µg g^−1^ (Fig. 6A).

The combined microcystin concentration was closely correlated with *M. aeruginosa* abundance (r = 0.93) within all the sampling months.

### Activity of the antioxidant system in *Daphnia* tissues

The average values of GSH, LPO, CAT differed significantly among animals from the three sites in each month studied (one-way ANOVA, details on Figs. 2B, C, D–5B, C, D). Significant differences for all the studied parameters were also observed in 2014 (one-way ANOVA, Fig. 6 B, C, D, E).

In June, the glutathione concentrations in the tissues of daphniids were 2.15 nmol GSH/mg protein at TR and 0.94 at ZA (Fig. 2B). In July, the GSH values reached 2.39 (TR), 3.82 (BR) and 3.33 nmol GSH/mg protein at ZA (Fig. 3B). Lower concentrations of glutathione were found in August: 1.66 (TR), 1.75 (BR) and 2.17 (ZA) nmol GSH/mg protein (Fig. 4B). In September, the highest GSH concentration was detected at BR (3.91 nmol GSH/mg protein), and it was comparable to those found at TR and ZA (2.24 and 1.94 nmol GSH/mg protein, respectively) (Fig. 5B). The lipid peroxidation of daphniid cells was the lowest in June (0.10 nmol MDA/mg protein at TR and 0.23 nmol MDA/mg protein at ZA) and highest in July (0.87 (TR), 0.89 (BR) and 1.83 nmol MDA/mg protein at ZA (Figs. 2C, 3C). In August, the lipid peroxidation reached a medium level: 0.34 (TR), 0.53 (BR) and 0.93 nmol MDA/mg protein at ZA (Fig. 4C). More diverse values of LPO were observed in September (0.28 at TR, 1.23 at BR and 0.72 nmol MDA/mg protein at ZA) (Fig. 5C). Similar to the LPO, the catalase activity was the lowest in June (3.58 at TR and 1.42 U/mg protein at ZA) and the highest in July (7.57 (TR), 24.20 (BR) and 22.81 U/mg protein at ZA) (Figs. 2D, 3D). In August (Fig. 4D), the values of CAT were as follows: 5.22 (TR), 6.47 (BR) and 8.45 U/mg protein (ZA). In September, the catalase activity reached 6.16 (TR), 12.31 (BR) and 4.81 U/mg protein at ZA (Fig. 5D).

The two-way ANOVA indicated a significant seasonal difference in the values of all the studied antioxidant parameters in *Daphnia* (Table 2). The values of GSH were the highest in June and lowest in September. The same pattern was observed in the case of the lipid peroxidation values and catalase activity. Differences between sites were considerable in all the months studied. However, as there were only two sites sampled in June, a two-way ANOVA was used for the July, August and September data analyses. Statistical analysis showed that values of GSH, LPO and CAT were always significantly higher in the TR station than in the BR and ZA stations. The month × site interactions in the antioxidant system parameters were significant and resulted from high LPO values at BR in September and low CAT values at both ZA in September and TR in July. In the case of glutathione, the month × site interaction was also significant and was largely an effect of the low GSH concentration both at ZA in September and BR in August (Table 2).

The results from September 2014 showed the same pattern of antioxidant system parameters activity as in the 2012 season. The lowest concentration of GSH (0.35 nmol GSH/mg protein) was observed in TR. The GSH concentrations at the other stations amounted to the following: 1.91 nmol GSH/mg protein in BR and 2.47 nmol GSH/mg protein in ZA (Fig. 6B). The GST activity was the highest in TR (83.46 U/mg protein) compared to BR (72.94 U/mg protein) and ZA (60.09 U/mg protein) (Fig. 6C). The values of LPO were lower in TR and ZA (0.087 and 0.074 nmol MDA/mg protein, respectively) than in BR (0.126 nmol MDA/mg protein) (Fig. 6D). The CAT activity was the lowest in TR (8.36 U/mg protein) and the highest in BR (17.81 U/mg protein) (Fig. 6E).

## Discussion

The spatial distribution of *M. aeruginosa* was not homogenous in the Sulejow Reservoir. The lacustrine part of the reservoir, which is below ZA, is characterised by physical and chemical parameters favouring cyanobacterial development: relatively stable (flood-free) hydrological conditions with retention times of up to 60 days and a high supply of nutrients from the catchment area [37]. In addition, the presence of numerous bays, i.e., shallow and wind-protected areas in this part of the reservoir, may be crucial for the recruitment of cyanobacteria from sediments (Izydorczyk, unpublished data). Moreover, the Sulejow Reservoir is geographically oriented from the southwest to northeast, whereas the winds in this area blow predominantly from the west and southwest. This means that wind moves blooms towards the dam [38], and therefore the TR station is characterised by the highest cyanobacterial concentrations, as confirmed by our results.

In 2012, the microcystin concentrations were closely correlated with cyanobacteria abundance, leading to spatial differences in the toxicity. However, the densities of *D. longispina* were comparable at all of the studied stations in a given month (Table 1), which indicates that the distribution of daphniids was not related to differences in the distribution of cyanobacteria. Nevertheless, we expected differences in the antioxidant system activity of *D. longispina* depending on the intensity of blooms. Indeed, the similar results achieved in both 2012 and 2014 indicate the occurrence of a relationship between the spatial diversity of cyanobacteria abundance and the effectiveness of the system that protects *Daphnia* against oxidative stress.

Microcystins, the main group of cyanotoxins, can induce oxidative stress in the cells of aquatic animals [39], which is related to the production of reactive oxygen species (ROS) and leads to an increase in lipid peroxidation [40], [41]. It is worth noting that lipid peroxidation is considered to be the major mechanism by which oxyradicals can cause tissue damage, impair cellular function and disrupt the physicochemical properties of cell membranes [42]. In our study, the oxidative stress of *D. longispina* caused by MCs was determined by the TBARS assay, reacting mainly with malondialdehyde (MDA) as the principal product from lipid peroxidation. Intriguingly, the results indicated the lowest level of LPO at the TR site, which was characterised by the highest *M. aeruginosa* abundance and toxicity in all studied months (Fig. 2C–5C). The high levels of LPO (also corresponding to high CAT activity) occurred at sites with a minimum biomass of cyanobacteria – mainly at ZA in July and August (Fig. 3A, C, D–4A, C, D). A possible explanation may be that daphniids from upper sites were less susceptible to cyanotoxins and thus had a less resistant antioxidant system, which responded more rapidly to biotic and abiotic environmental factors. The CAT activity increased and hence mitigated oxidative damage, which is why we observed elevated values of CAT when LPO reached high levels (Fig. 3C, D–5C, D). This would confirm the finding that, when faced with an increasing production of ROS resulting from the effects of cyanotoxins, organisms usually up-regulate antioxidant defences such as catalase activity [43]. However, the low CAT activity at TR indicates that the main defence mechanism of *Daphnia* in the presence of toxic blooms was instead the process of microcystin detoxification. This may also be supported by the low glutathione concentration at TR, as glutathione can be used for the production of MC-GSH conjugates [17]. The additional measurement of the glutathione S-transferase activity in September 2014 confirmed this conclusion. The high GST activity corresponded with low GSH concentration at the TR station, which indicates the most intensive detoxification at the site with the highest toxic thread (Fig. 6C, D). The chemical conjugation of MCs with glutathione is recognised as the first step of detoxification in aquatic organisms exposed to cyanobacterial toxins because production of conjugates reduces the toxicity of MCs and facilitates their excretion by organisms [44]. This ability of glutathione confirms its particularly important function in diminishing oxidative stress [45].

Our results corroborate the conclusions of Lemaire et al. [12] that *Daphnia* do not develop generalised responses against *Microcystis* but rather specifically adapt to local assemblages of toxic cyanobacteria strains. Additionally, our study indicates that such adaptation also appears within an ecosystem with a different spatial distribution of blooms. These new facets of the *Daphnia-Microcystis* interaction may be crucial for the stabilisation of the top-down effect of grazers in the trophic structure, for the limitation of microcystin accumulation by *Daphnia* and thus for the reduction of the contribution of daphniids to the transfer of toxins to higher trophic levels in food webs [6], [9], [46].

## Supporting Information

Table S1 **The data represent three/seven replicates (1–7), mean and standard deviation (SD) of glutathione concentration (nmol/mg protein) in** ***Daphnia*** **tissues from the Sulejow Reservoir.**
(DOCX)

Table S2 **The data represent three/seven replicates (1–7), mean and standard deviation (SD) of lipid peroxidation (nmol/mg protein) in** ***Daphnia*** **tissues from the Sulejow Reservoir.**
(DOCX)

Table S3 **The data represent three/six replicates (1–6), mean and standard deviation (SD) of catalase activity (U/mg protein) in** ***Daphnia*** **tissues from the Sulejow Reservoir.**
(DOCX)

Table S4 **The data represent three replicates (1–3), mean and standard deviation (SD) of glutathione S-transferase activity (U/mg protein) in** ***Daphnia*** **tissues from the Sulejow Reservoir.**
(DOCX)
