# Supplementary material for: Response of Daphnia's Antioxidant System to Spatial Heterogeneity in Cyanobacteria Concentrations in a Lowland Reservoir
Source: PLoS One. 2014 Nov 7;9(11):e112597. doi: 10.1371/journal.pone.0112597 (PMC4224506; doi:10.1371/journal.pone.0112597)
Supplement: Table S1 — The data represent three/seven replicates (1–7), mean and standard deviation (SD) of glutathione concentration (nmol/mg protein) in Daphnia tissues from the Sulejow Reservoir. (DOCX) [file pone.0112597.s001.docx]

**Supporting table S1. The data represent three/seven replicates (1-7), mean and standard deviation (SD) of glutathione concentration (nmol/mg protein) in *Daphnia* tissues from the Sulejow Reservoir.**

Study sites: Tresta (TR), Bronisławów (BR) and Zarzęcin (ZA).

| Date | Site | 1 | 2 | 3 | 4 | 5 | 6 | 7 | Mean | SD |
| --- | --- | --- | --- | --- | --- | --- | --- | --- | --- | --- |
| 04.06.2012 | TR | 2.381 | 2.361 | 2.063 | 2.689 | 1.631 | 1.784 | 2.161 | **2.153** | 0.364 |
| 04.06.2012 | ZA | 1.014 | 0.826 | 0.801 | 1.322 | 0.431 | 0.966 | 1.230 | **0.942** | 0.296 |
| 02.07.2012 | TR | 2.526 | 1.770 | 2.013 | 1.942 | 2.934 | 2.401 | 3.160 | **2.393** | 0.521 |
| 02.07.2012 | BR | 3.268 | 4.059 | 3.877 | 3.900 | 3.611 | 4.410 | 3.640 | **3.824** | 0.364 |
| 02.07.2012 | ZA | 4.203 | 3.378 | 3.029 | 3.292 | 3.197 | 3.250 | 2.937 | **3.326** | 0.415 |
| 21.08.2012 | TR | 1.674 | 1.808 | 1.147 | 1.823 | 1.649 | 1.532 | 1.956 | **1.656** | 0.263 |
| 21.08.2012 | BR | 1.435 | 1.590 | 1.676 | 1.939 | 1.891 | 1.878 | 1.842 | **1.750** | 0.187 |
| 21.08.2012 | ZA | 1.862 | 2.294 | 2.319 | 1.643 | 2.166 | 2.760 | 2.136 | **2.168** | 0.355 |
| 26.09.2012 | TR | 2.315 | 2.448 | 2.279 | 2.089 | 2.339 | 1.857 | 2.331 | **2.237** | 0.199 |
| 26.09.2012 | BR | 4.836 | 4.047 | 3.633 | 3.351 | 3.800 | 3.688 | 4.021 | **3.911** | 0.472 |
| 26.09.2012 | ZA | 1.877 | 1.672 | 1.834 | 1.692 | 2.220 | 2.177 | 2.110 | **1.940** | 0.227 |
| 11.09.2014 | TR | 0.351 | 0.369 | 0.325 | - | - | - | - | **0.350** | 0.020 |
| 11.09.2014 | BR | 1.575 | 2.303 | 1.853 | - | - | - | - | **1.910** | 0.371 |
| 11.09.2014 | ZA | 2.137 | 2.211 | 3.056 | - | - | - | - | **2.470** | 0.510 |
