# Supplementary material for: Response of Daphnia's Antioxidant System to Spatial Heterogeneity in Cyanobacteria Concentrations in a Lowland Reservoir
Source: PLoS One. 2014 Nov 7;9(11):e112597. doi: 10.1371/journal.pone.0112597 (PMC4224506; doi:10.1371/journal.pone.0112597)
Supplement: Table S2 — The data represent three/seven replicates (1–7), mean and standard deviation (SD) of lipid peroxidation (nmol/mg protein) in Daphnia tissues from the Sulejow Reservoir. (DOCX) [file pone.0112597.s002.docx]

**Supporting table S2. The data represent three/seven replicates (1-7), mean and standard deviation (SD) of lipid peroxidation (nmol/mg protein) in *Daphnia* tissues from the Sulejow Reservoir.**

| Date | Site | 1 | 2 | 3 | 4 | 5 | 6 | 7 | Mean | SD |
| --- | --- | --- | --- | --- | --- | --- | --- | --- | --- | --- |
| 04.06.2012 | TR | 0.071 | 0.072 | 0.059 | 0.116 | 0.117 | 0.125 | 0.124 | **0.098** | 0.028 |
| 04.06.2012 | ZA | 0.215 | 0.228 | 0.237 | 0.239 | 0.259 | 0.201 | 0.212 | **0.227** | 0.019 |
| 02.07.2012 | TR | 0.804 | 0.747 | 0.720 | 0.732 | 1.047 | 1.065 | 1.006 | **0.875** | 0.157 |
| 02.07.2012 | BR | 1.180 | 1.180 | 1.159 | 0.737 | 0.677 | 0.640 | 0.656 | **0.890** | 0.266 |
| 02.07.2012 | ZA | 2.077 | 2.088 | 2.237 | 1.661 | 1.590 | 1.570 | 1.554 | **1.825** | 0.295 |
| 21.08.2012 | TR | 0.321 | 0.326 | 0.334 | 0.358 | 0.310 | 0.369 | 0.332 | **0.336** | 0.020 |
| 21.08.2012 | BR | 0.530 | 0.509 | 0.960 | 0.459 | 0.422 | 0.410 | 0.404 | **0.528** | 0.196 |
| 21.08.2012 | ZA | 1.150 | 1.150 | 0.549 | 0.910 | 0.879 | 0.891 | 0.955 | **0.926** | 0.202 |
| 26.09.2012 | TR | 0.307 | 0.294 | 0.299 | 0.328 | 0.249 | 0.247 | 0.258 | **0.283** | 0.031 |
| 26.09.2012 | BR | 1.200 | 1.188 | 1.225 | 1.305 | 1.146 | 1.263 | 1.252 | **1.226** | 0.052 |
| 26.09.2012 | ZA | 0.666 | 0.843 | 0.783 | 0.757 | 0.716 | 0.677 | 0.598 | **0.720** | 0.081 |
| 11.09.2014 | TR | 0.086 | 0.088 | 0.088 | - | - | - | - | **0.087** | 0.001 |
| 11.09.2014 | BR | 0.131 | 0.111 | 0.136 | - | - | - | - | **0.126** | 0.013 |
| 11.09.2014 | ZA | 0.081 | 0.071 | 0.069 | - | - | - | - | **0.074** | 0.007 |

Study sites: Tresta (TR), Bronisławów (BR) and Zarzęcin (ZA).
