# Supplementary material for: Response of Daphnia's Antioxidant System to Spatial Heterogeneity in Cyanobacteria Concentrations in a Lowland Reservoir
Source: PLoS One. 2014 Nov 7;9(11):e112597. doi: 10.1371/journal.pone.0112597 (PMC4224506; doi:10.1371/journal.pone.0112597)
Supplement: Table S3 — The data represent three/six replicates (1–6), mean and standard deviation (SD) of catalase activity (U/mg protein) in Daphnia tissues from the Sulejow Reservoir. (DOCX) [file pone.0112597.s003.docx]

| Date | Site | 1 | 2 | 3 | 4 | 5 | 6 | Mean | SD |
| --- | --- | --- | --- | --- | --- | --- | --- | --- | --- |
| 04.06.2012 | TR | 3.84 | 3.51 | 3.58 | 3.59 | 3.31 | 3.65 | **3.58** | 0.172 |
| 04.06.2012 | ZA | 1.91 | 1.46 | 1.16 | 1.14 | 1.61 | 1.25 | **1.42** | 0.301 |
| 02.07.2012 | TR | 7.39 | 7.90 | 7.74 | 7.47 | 7.25 | 7.69 | **7.57** | 0.243 |
| 02.07.2012 | BR | 24.20 | 24.77 | 20.04 | 26.31 | 24.20 | 25.66 | **24.20** | 2.201 |
| 02.07.2012 | ZA | 21.04 | 23.09 | 23.83 | 23.60 | 22.15 | 23.13 | **22.81** | 1.039 |
| 21.08.2012 | TR | 5.83 | 4.89 | 5.05 | 4.87 | 5.20 | 5.50 | **5.22** | 0.377 |
| 21.08.2012 | BR | 6.97 | 6.38 | 6.21 | 6.73 | 6.00 | 6.55 | **6.47** | 0.352 |
| 21.08.2012 | ZA | 9.54 | 7.77 | 8.83 | 8.37 | 7.48 | 8.71 | **8.45** | 0.748 |
| 26.09.2012 | TR | 6.56 | 6.11 | 6.13 | 5.96 | 6.12 | 6.07 | **6.16** | 0.207 |
| 26.09.2012 | BR | 10.59 | 13.26 | 12.29 | 12.70 | 12.03 | 13.01 | **12.31** | 0.958 |
| 26.09.2012 | ZA | 4.20 | 4.75 | 5.05 | 5.12 | 4.82 | 4.92 | **4.81** | 0.329 |
| 11.09.2014 | TR | 8.71 | 8.76 | 7.60 | - | - | - | **8.36** | 0.656 |
| 11.09.2014 | BR | 16.91 | 17.80 | 18.72 | - | - | - | **17.81** | 0.903 |
| 11.09.2014 | ZA | 15.40 | 14.55 | 13.29 | - | - | - | **14.41** | 1.060 |

**Supporting table S3. The data represent three/six replicates (1-6), mean and standard deviation (SD) of catalase activity (U/mg protein) in *Daphnia* tissues from the Sulejow Reservoir.**

Study sites: Tresta (TR), Bronisławów (BR) and Zarzęcin (ZA).
