# Supplementary material for: Response of Daphnia's Antioxidant System to Spatial Heterogeneity in Cyanobacteria Concentrations in a Lowland Reservoir
Source: PLoS One. 2014 Nov 7;9(11):e112597. doi: 10.1371/journal.pone.0112597 (PMC4224506; doi:10.1371/journal.pone.0112597)
Supplement: Table S4 — The data represent three replicates (1–3), mean and standard deviation (SD) of glutathione S-transferase activity (U/mg protein) in Daphnia tissues from the Sulejow Reservoir. (DOCX) [file pone.0112597.s004.docx]

**Supporting table S4. The data represent three replicates (1-3), mean and standard deviation (SD) of glutathione S-transferase activity (U/mg protein) in *Daphnia* tissues from the Sulejow Reservoir.**

| Date | Site | 1 | 2 | 3 | Mean | SD |
| --- | --- | --- | --- | --- | --- | --- |
| 11.09.2014 | TR | 85.33 | 84.74 | 80.30 | **83.46** | 2.75 |
| 11.09.2014 | BR | 72.01 | 75.76 | 71.04 | **72.94** | 2.49 |
| 11.09.2014 | ZA | 67.19 | 57.54 | 55.52 | **60.09** | 6.24 |

Study sites: Tresta (TR), Bronisławów (BR) and Zarzęcin (ZA).
